# Promoting Effect and Potential Mechanism of *Lactobacillus pentosus* LPQ1-Produced Active Compounds on the Secretion of 5-Hydroxytryptophan

**DOI:** 10.3390/foods11233895

**Published:** 2022-12-02

**Authors:** Yixiu Zeng, Jiajia Song, Yuhong Zhang, Yechuan Huang, Feng Zhang, Huayi Suo

**Affiliations:** 1 College of Food Science, Southwest University, Chongqing 400715, China; 2 College of Bioengineering, Jingchu University of Technology, Jingmen 448000, China; 3 Institute of Food Sciences and Technology, Tibet Academy of Agricultural and Animal Husbandry Sciences, Lhasa 850000, China; 4 Chongqing Tianyou Dairy Co., Ltd., Chongqing 401120, China

**Keywords:** 5-hydroxytryptophan, lactic acid bacteria, depression, RIN-14B cells

## Abstract

5-hydroxytryptophan (5-HTP) is an important substance thought to improve depression. It has been shown that Lactobacillus can promote the secretion of 5-HTP in the body and thus ameliorate depression-like behavior in mice. However, the mechanism by which Lactobacillus promotes the secretion of 5-HTP is unclear. In this study, we investigated the promoting effect and mechanism of Lactobacillus, isolated from Chinese fermented foods, on the secretion of 5-HTP. The results showed that *Lactobacillus (L.) pentosus* LPQ1 exhibited the strongest 5-HTP secretion-promoting effect ((9.44 ± 0.69)-fold), which was dependent on the mixture of compounds secreted by *L. pentosus* LPQ1 (termed SLPQ1). In addition, the results of the RNA sequencing (RNA-seq) and quantitative real-time polymerase chain reaction (qRT-PCR) analyses indicated that SLPQ1 alters the TNF and oxidative phosphorylation signaling pathways. Moreover, the SLPQ1 ultrafiltration fraction (>10 kDa) showed a similar 5-HTP promoting effect as SLPQ1. Furthermore, reverse-phase liquid chromatography-tandem mass spectrometry (RPLC-MS/MS) identified 29 compounds of >10 kDa in SLPQ1, including DUF488 domain-containing protein, BspA family leucine-rich repeat surface protein, and 30S ribosomal protein S5, which together accounted for up to 62.51%. This study reports new findings on the mechanism by which *L. pentosus* LPQ1 promotes 5-HTP production in some cell lines in vitro.

## 1. Introduction

Depression is a major contributor to disability and also imposes a significant economic and health care burden on patients, their families, and the health system [1,2]. Experts predict that by 2030, the cost of curing mental illnesses, including depression, will be as high as $6 trillion [3]. Antidepressants, such as the selective 5-hydroxytryptamine (5-HT), also known as serotonin reuptake inhibitor fluoxetine, have antidepressant effects by blocking 5-HT reuptake [4]. However, patients take antidepressant medications for a long time before they relieve depression, and they are often accompanied by adverse side effects [5,6]. Food-derived substances without side effects have been reported to alleviate depression by improving neurotransmitter synthesis, modulating the activation of the brain-derived neurotrophic factor tropomyosin receptor kinase B (BDNF-TrκB) pathway, inhibiting the expression of inflammatory genes, and reshaping the intestinal flora of depressed mice [7,8]. Therefore, safe food-derived substances are thought to have the potential to improve depression.

Insufficient levels of synaptic 5-HT in the brain are considered to be a manifestation of the classical pathogenesis of depression [9]. The neurotransmitter 5-HT ameliorates depression mainly by acting on mood-related nervous systems [10]. However, 5-HT cannot cross the blood-brain barrier (BBB) and is synthesized locally in the brain from its precursors [11]. Both tryptophan and 5-hydroxytryptophan (5-HTP) are used in the biosynthesis of 5-HT. Roughly 90% of dietary tryptophan is used in the kynurenine pathway, a process that competes with the synthetic pathway of 5-HT [12,13]. Unlike tryptophan, 5-HTP freely crosses the BBB and increases the 5-HT levels in the brain to exert an antidepressant effect [14,15,16]. In 30 patients with their first depressive episode treated with 5-HTP for two weeks, 5-HTP showed some antidepressant effect in 73.33% of the patients [17]. Similarly, 40 out of 59 patients with depressive symptoms who were given 150–300 mg of 5-HTP daily for three weeks showed significant improvement in depressive symptoms [18]. However, oral 5-HTP has been reported to cause adverse side effects, such as abnormal psychological functioning, behavioral disturbances and intolerance [15].

Lactic acid bacteria (LAB) derived from fermented foods are considered natural and safe [19]. Accordingly, the use of probiotics for the alleviation of neurodegenerative diseases, including depression, have gained attention in recent years [20]. The relative abundance of Lactobacillus was reported to be closely related to the serum level of 5-HTP [21]. *Lactococcus lactis* WHH2078 was also found to modulate 5-HT metabolism by increasing colonic and serum 5-HTP levels, thus exerting antidepressant effects in mice [22]. Similarly, Bifidobacterium exhibiting 5-HTP synthesis modulating effects have been found to alleviate depressive symptoms and associated microbiota dysbiosis in mice [23]. However, the mechanisms by which LAB enhance 5-HTP secretion have not been reported.

In this study, we evaluated the promotion of 5-HTP secretion in RIN-14B cells by ten LAB strains from traditional Chinese fermented foods and investigated the active components and mechanism of action of the LAB strain with the highest 5-HTP-promoting effect. To the best of our knowledge, this is the first report of the mechanism by which active compounds secreted by LAB strains regulate the secretion of 5-HTP in RIN-14B cells.

## 2. Materials and Methods

### 2.1. Materials and Reagents

*Lactobacillus* (*L.*) *pentosus* LPQ1 and *L. helveticus* Q13 were from Qula, a raw yak milk cheese (Qinghai, China). *L. plantarum* M45, *L. casei* 96, *L. plantarum* 05015, *L. plantarum* Q11, and *L. plantarum* Q9 were from traditional fermented pickles (Chongqing, China). *L. rhamnosus* T53 and *L. helveticus* Y4 were from traditional fermented yogurt (Qinghai, China). *L. plantarum* 05007 was from traditional fermented yogurt (Chengdu, China). Hanks’ balanced salt solution (HBSS) and RNase-free water were obtained from Beyotime Biotechnology Inc. (Shanghai, China). Fetal bovine serum (FBS) was supplied by Biological Industries Beit HaEmek Ltd. (Beit HaEmek, Israel). TRIzol reagent was obtained from Thermo Fisher Scientific Inc. (Waltham, MA, USA). RPMI1640 medium was purchased from Beijing Solarbio Technology Co., Ltd. (Beijing, China).

### 2.2. RIN-14B Cell Culture

RIN-14B cells were provided by American Typical Culture Collection (ATCC, Manassas, VA, USA). RIN-14B cells were grown in RPMI 1640 with 10% FBS, 1% penicillin-streptomycin solution, in an incubator at 37 °C with a humidified atmosphere and 5% CO_2_. After reaching confluence of approximately 80%, cells were detached by digestion with trypsin and passaged. The culture medium was changed every 2–3 days. RIN-14B cells from the 10th to 30th generation were used in this study.

### 2.3. The Preparation of LAB Strains

The preparation of LAB was performed according to a previous study [19] with slight modifications. The LAB strains were inoculated (2%, *v*/*v*) in de Man-Rogosa-Sharpe (MRS) medium and then incubated at 37 °C for 18 h. After centrifuging at 4000× *g* for 10 min at 4 °C, the bacteria were collected, and then the number of bacterial cells was adjusted to 1 × 10^9^ CFU/mL.

### 2.4. Preparation of the Mixture of Compounds Secreted by L. pentosus LPQ1 (SLPQ1)

*L. pentosus* LPQ1 at 1 × 10^9^ CFU/mL was incubated in an HBSS solution for 30 min at 37 °C and centrifuged at 4000× *g* for 10 min at 4 °C. Afterwards, the supernatant was collected and then filtered through a 0.22-μL filter to obtain the mixture of secreted compounds. The mixture of compounds secreted by *L. pentosus* LPQ1 (termed SLPQ1) was lyophilized and stored at −80 °C for subsequent studies.

### 2.5. Evaluation of the Promoting Effect of LAB and Its Secreted Compounds on the Secretion of 5-HTP by RIN-14B Cells

As described previously, we performed experiments on the amount of 5-HTP secretion [24,25]. RIN-14B cells were inoculated into 24-well plates at a density of 4 × 10^5^ cells/mL and incubated for 72 h. Then, after washing the cells with 1 mL of HBSS buffer with 0.1% bovine serum albumin (BSA) and 2 M fluoxetine, 1 mL of an HBSS suspension containing LAB or their secreted compounds was added to the experimental group, while only the HBSS solution without LAB or their secretions was added to the control group. After incubation for 30 min at 37 °C, the supernatants were collected and centrifuged to remove the precipitate.

### 2.6. Determination of 5-hydroxytryptophan Content

The test method for the measurement of the 5-HTP content was based on a previously reported method [24]. In brief, the cell supernatant was passed through a 0.22-μM filter. The 5-HTP content of the cell supernatant was assayed using a HPLC system (Shimadzu Corporation, Kyoto, Japan) equipped with a fluorescence detector (RF-20A, Shimadzu). The 20 μL of sample was loaded on an Agilent Zorbax XDB-C18 column (250 mm × 4.6 mm, 5 μm), and eluted with 0.1 M sodium acetate buffer (containing 0.1 mM EDTA-2Na) and methanol in a ratio of 85:15 (*v*/*v*). The column temperature was 30 °C, and the flow rate was 1.0 mL/min. The fluorescence intensity was detected at wavelengths of 330 nm (emission) and 290 nm (excitation).

### 2.7. Methyl Thiazolyl Tetrazolium (MTT) Assay

The MTT assay was performed as described previously [26], with slight modifications. RIN-14B cells were seeded into 96-well plates at a density of 4 × 10^5^ cells/mL and grown for 72 h. Subsequently, after gently aspirating the medium and washing the cells once with HBSS, 100 μL of sample solution was added to each well and the plate was incubated for 30 min. Afterwards, 10 μL of MTT reagent was added to each well, and the plate was protected from light and incubated at 37 °C for 4 h. Then, after removing the supernatant from all wells, 100 L of dimethyl sulfoxide (DMSO) was added to each well, and after agitating the mixture for 15 min the absorbance (Abs) of each well was measured at 490 nm. The cell viability was estimated using the formula:

Cell viability (%) = (Abs _sample_ − Abs _blank_) × 100/(Abs _control_ − Abs _blank_).

### 2.8. RNA-Seq Analysis

RIN-14B cells were inoculated into 24-well plates at a density of 4 × 10^5^ cells/mL and then incubated for 72 h. Subsequently, after discarding the medium and washing the cells with 1 mL of HBSS buffer containing 0.1% BSA and 2 μM fluoxetine, 1 mL of HBSS suspension containing 16 mg/mL of SLPQ1 was added to each well (the control group received HBSS solution without SLPQ1). After incubation at 37 °C for 30 min, the supernatant was gently aspirated and discarded. Then, after washing the attached cells with pre-cooled HBSS, the cells were lysed by adding 1 mL of TRIzol reagent, and the cell lysate was transferred into sterile enzyme-free lyophilization tubes, snap-frozen in liquid nitrogen and stored at −80 °C for subsequent RNA extractions. The samples were subjected to RNA-seq analysis using a previously described procedure [27]. In brief, total cellular RNA was extracted using TRIzol (Thermo Fisher Scientific, Waltham, MA, USA). The purity and concentration of the extracted RNA were assayed using a NanoDrop 2000 spectrophotometer (Thermo Fisher Scientific), and its integrity was assessed by agarose gel electrophoresis. Afterwards, the mRNA was enriched from total RNA using oligo dT affinity beads and then randomly split into 300 bp fragments. These fragments were used as templates for reverse transcription and the production of double-stranded cDNA. A single nucleotide A and a connector were connected to the end-repaired double-stranded cDNA, following PCR amplification for the de novo RNA-seq cDNA library. Sequencing was performed using the NovaSeq 6000 System (Illumina, Inc., San Diego, CA, USA), and the bioinformatics analysis was performed using the free online platform of MajorBio Cloud Platform (https://cloud.majorbio.com, accessed on 18 October 2022).

### 2.9. qRT-PCR Analysis

Total RNA was extracted using TRIzol reagent, and subsequently its concentration and purity were determined. Then, total RNA was reverse transcribed into cDNA using a cDNA reverse transcription kit (Thermo Fisher Scientific Inc.) and stored at −80 °C for subsequent analysis. PCR reaction conditions were as follows: pre-denaturation at 95 °C for 10 min; followed by 40 cycles at 95 °C, 15 s; 60 °C, 1 min; 72 °C, 30 s. The 2^−ΔΔCT^ method was used to calculate the expression of the target gene primer sequences, which are shown in Table S1.

### 2.10. Analysis of Active Compounds

Appropriate amounts of samples were separated using 10 kDa, 3 kDa and 1 kDa ultrafiltration tubes. The recovered fractions were then lyophilized and kept at −80 °C for further studies. The reverse-phase liquid chromatography-tandem mass spectrometry (RPLC-MS/MS) analysis of the fraction of >10 kDa was carried out according to a previously reported method [28], with some modifications. Briefly, the fraction of >10 kDa was denatured with 8 M urea, reduced with 10 mM iodoacetamide, and then alkylated with 20 mM iodoacetamide. Subsequently, the trypsin (Promega, WI, USA) at an enzyme/substrate ratio of 1:50 (*w*/*w*) was added for digestion at 37 °C for 18 h. The digested sample was desalted with C18 ZipTips microcolumns (Millipore, Burlington, MA, USA) and then lyophilized for further RPLC-MS/MS analysis.

The lyophilized sample was in 0.1% (*v*/*v*) formic acid and was analyzed by a QE-HF mass spectrometer (Thermo Fisher Scientific, Waltham, MA, USA) coupled to an Easy-nLC 1000 HPLC system (Thermo Fisher Scientific). The sample was loaded onto a C18 reversed-phase analytical column (Acclaim PepMap RSLC, 75 µm × 15 cm, nanoViper, 2 µm 100 Å C18 particles; Thermo Fisher Scientific) and eluted with a linear gradient of 0.1% (*v*/*v*) formic acid and 0.1% (*v*/*v*) formic acid in 80% (*v*/*v*) acetonitrile for 70 min at a flow rate of 400 nL/min. The MS1 scan was acquired in the mass range of 350–1550 *m*/*z* at a resolution of 120,000, with an automatic gain control (AGC) target value of 4 × 10^5^ and a maximum injection time (IT) of 50 ms. The MS2 scan was performed at a resolution of 30,000 with an AGC target value of 1e5, a maximum IT of 100 ms, and a normalized collision energy of 32 eV. The raw MS files were analyzed using PEAKS Studio v7.5 software (Bioinformatic Solutions Inc, waterloo, ON, Canada).

### 2.11. Statistical Analysis

The data from each test, performed at least three times, were expressed as the mean standard deviation. All data were evaluated for statistically significant differences (*p* < 0.05) by one-way analysis of variance (ANOVA) with Duncan’s test using the IBM SPSS 23 software (IBM Corporation, Armonk, NY, USA).

## 3. Results

### 3.1. Effect of LAB Strains on 5-HTP Secretion

**Figure 1:**
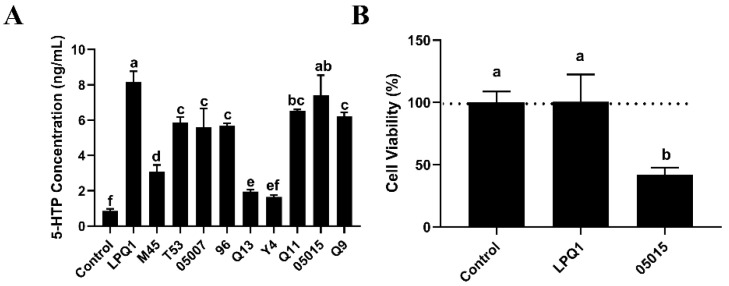
Effects of different lactic acid bacteria (LAB) strains on the secretion of 5-hydroxytryptophan (5-HTP) by RIN-14B cells. (**A**) Effects of LAB strains on the secretion of 5-HTP by RIN-14B cells. (**B**) Cell viability. Control: RIN-14B cells; LPQ1: RIN-14B cells with *Lactobacillus (L.) pentosus* LPQ1; M45: RIN-14B cells with *L. plantarum* M45; T53: RIN-14B cells with *L. rhamnosus* T53; 05007: RIN-14B cells with *L. plantarum* 05007; 96: RIN-14B cells with *L. casei* 96; Q13: RIN-14B cells with *L. helveticus* Q13; Y4: RIN-14B cells with *L. helveticus* Y4; Q11: RIN-14B cells with *L. plantarum* Q11; 05015: RIN-14B cells with *L. plantarum* 05015; Q9: RIN-14B cells with *L. plantarum* Q9. Data are expressed as the mean ± standard deviation (*n* = 3). Different letters (a–f) indicate significant differences (*p* < 0.05).

As shown in Figure 1A, among ten LAB strains, the strains LPQ1 and 05015 exhibited a strong 5-HTP production and secretion effect on RIN-14B cells. However, as shown in Figure 1B, the 05015 strain was somewhat toxic to the cells; for this reason, the LPQ1 strain was used as a potential strain to increase the secretion of 5-HTP in subsequent experiments.

### 3.2. Effect of SLPQ1 on 5-HTP Secretion by RIN-14B Cells

**Figure 2:**
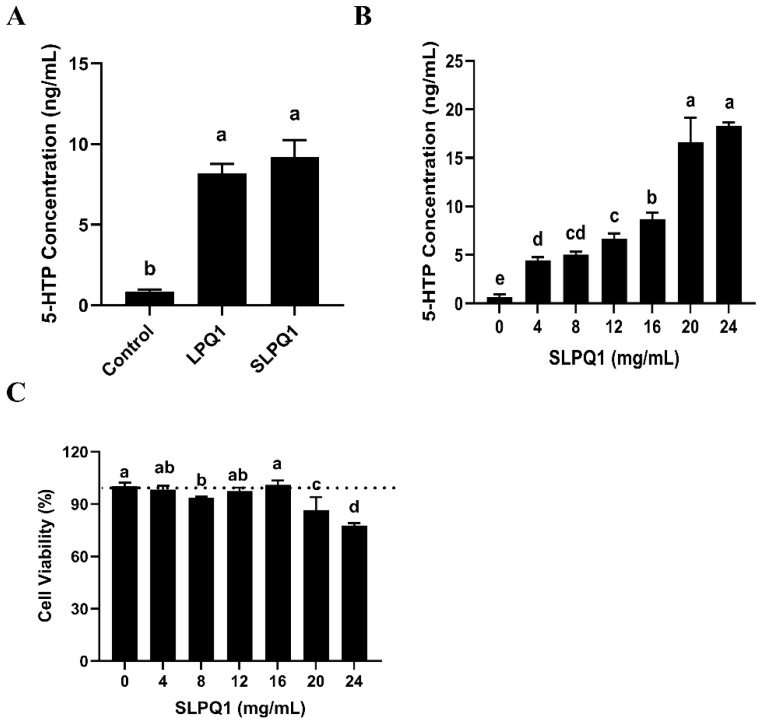
Effect of active ingredients of the mixture of secreted compounds, SLPQ1, collected from *Lactobacillus* (*L.*) *pentosus* LPQ1 on the secretion of 5-hydroxytryptophan (5-HTP). (**A**) Identification of effective compounds. (**B**) Determination of the amount of SLPQ1 used. (**C**) Effect of different SLPQ1 amounts on cell activity. Control: control group; LPQ1: treatment group after *L. pentosus* LPQ1; LPQ1-S: treatment group after secretion of *L. pentosus* LPQ1 without *L. pentosus* LPQ1 (no freeze-drying); SLPQ1: treatment group after SLPQ1. Data are expressed as the mean ± standard deviation (*n* = 3). Different letters (a–e) indicate significant differences (*p* < 0.05).

There was no significant difference between the amount of 5-HTP secreted by RIN-14B cells treated with *L. pentosus* LPQ1 and those treated with its secreted compounds SLPQ1, as shown in Figure 2A (*p* > 0.05). Therefore, the compound that affects the production and secretion of 5-HTP by RIN-14B cells may be a component of the SLPQ1 mixture. Indeed, SLPQ1 promoted 5-HTP secretion from RIN-14B cells in a concentration-dependent manner (Figure 2B). However, while 16 mg/mL SLPQ1 was the optimal concentration, SLPQ1 concentrations greater than 16 mg/mL had significant toxic effects on the cells (Figure 2C). These results suggested that the active ingredient of *L. pentosus* LPQ1 is among its secreted compounds.

### 3.3. Analysis of the Transcriptional Changes

We performed RNA-seq analysis on RIN-14B cells treated with SLPQ1 in order to understand the SLPQ1-mediated molecular regulation of 5-HTP production and secretion by RIN-14B cells. The total number of raw sequencing data entries generated per sample exceeded 60,000,000, with Q20 and Q30 exceeding 98 and 94%, respectively, as shown in Table S2.

**Figure 3:**
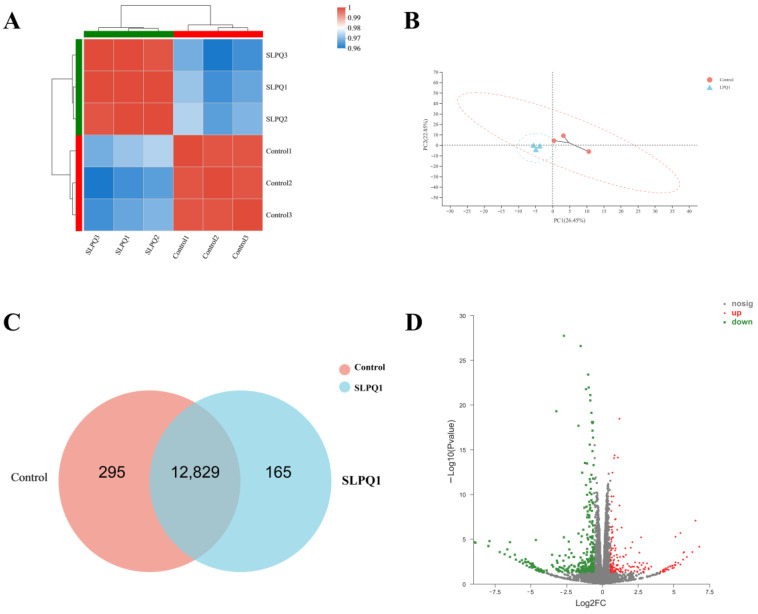
Effects of the mixture of secreted compounds, SLPQ1, collected from *Lactobacillus pentosus* LPQ1 on the global gene expression profiling in RIN-14B cells. (**A**) Sample correlation heatmap. (**B**) Principal component analysis (PCA). (**C**) Veen diagram. (**D**) Volcano plot. Control: control group; SLPQ1: treatment group after SLPQ1. Control: control group; SLPQ1: treatment group after SLPQ1.

Transcripts Per Million reads (TMPs) sequentially normalizes gene length and sequencing depth, a process that allows for consistent total expression between samples and thus comparison of expression between genes. Using the Pearson correlation coefficient, a measure of the linear correlation between two variables, the closer the R value is to 1, the stronger the correlation between the samples. Intra-group correlations were high for the control and SLPQ1 groups, but the inter-group correlations were low (Figure 3A). Principal component analysis (PCA) clustered the samples according to their expression level (high or low), and the higher the similarity of the samples, the closer the distribution of sample points. The control group and SLPQ1 group were closer within each group and further apart between the groups (Figure 3B). The Veen analysis can identify the number of genes co-expressed and specifically expressed between different groups. There are 295 genes specifically expressed in the control group and 165 genes specifically expressed in the SLPQ1 group, and a total of 12,829 genes expressed in both groups (Figure 3C). The volcano plot allowed visualization of upregulated or downregulated genes. As shown in Figure 3D, there were 485 changed genes, including 169 upregulated genes and 316 downregulated genes. These findings support the notion that SLPQ1 significantly affects functions of RIN-14B cells.

### 3.4. Differentially Expressed Genes Analysis by Gene Ontology Term and Kyoto Encyclopedia of Genes and Genomes Pathway Enrichment Analyses

**Figure 4:**
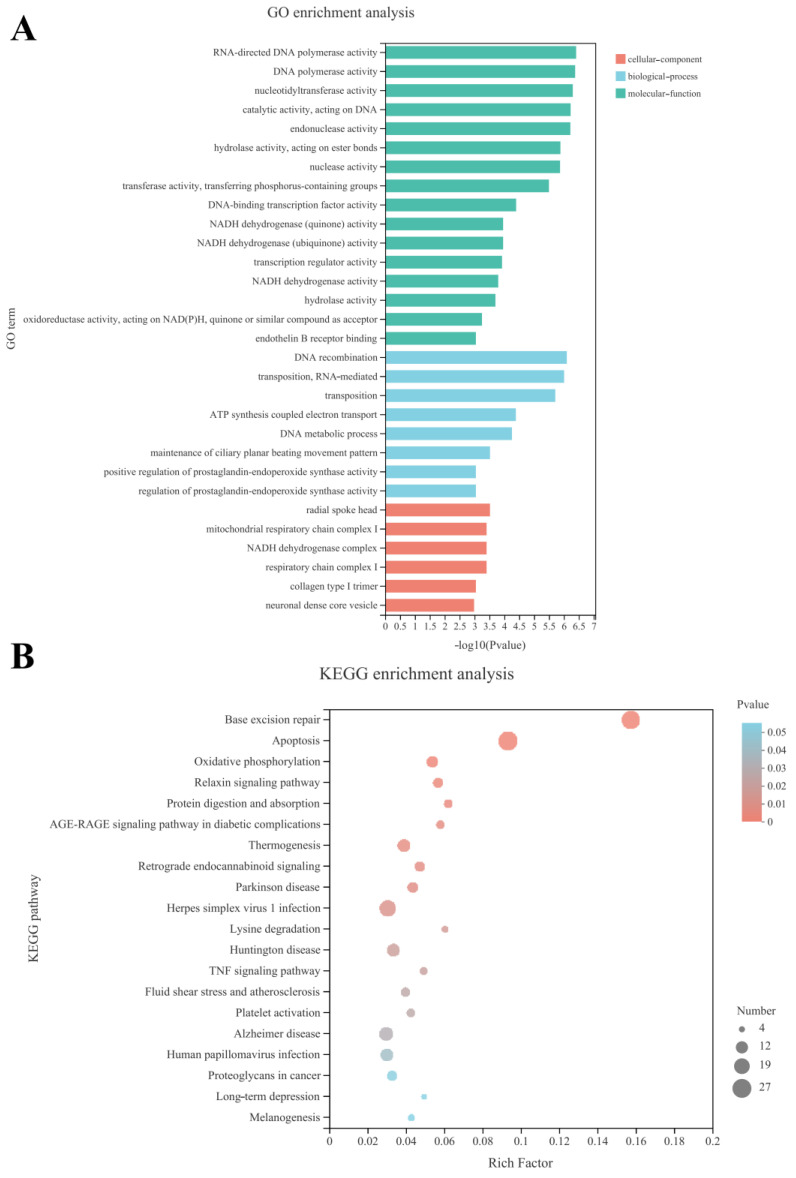
Effects of the mixture of secreted compounds, SLPQ1, collected from *Lactobacillus pentosus* LPQ1 on Gene Ontology (GO) term enrichment and Kyoto Encyclopedia of Genes and Genomes (KEGG) pathway enrichment analysis. (**A**) GO term enrichment analysis. (**B**) KEGG pathway enrichment analysis. Control: control group; SLPQ1: treatment group after SLPQ1.

The Gene Ontology (GO) database allows the qualification and description of genes, which are classified into three categories: biological process (BP), molecular function (MF) and cellular composition (CC) [19]. The top 30 enriched GO terms are shown in Figure 4A.

Significant functional differences between the SLPQ1 and Control groups were found in the following GO CC categories enriched with differentially expressed genes (DEGs): radial spoke head, mitochondrial respiratory chain complex I, NADH dehydrogenase complex, etc., as shown in Figure 4A. Significant functional differences between the SLPQ1 and Control groups were found in the following GO BP categories enriched with DEGs: DNA recombination, transposition and RNA-mediated, transposition, etc., as shown in Figure 4A. Additionally, RNA-directed DNA polymerase activity, DNA polymerase activity, and nucleotidyltransferase activity, etc., are among a few of the noteworthy functional changes between the SLPQ1 and Control groups in the GO MF enriched with DEGs (Figure 4A).

The Kyoto Encyclopedia of Genes and Genomes (KEGG) pathway enrichment analysis revealed the signaling pathways significantly enriched with DEGs/transcripts. The top 20 significantly enriched pathways identified by KEGG pathway enrichment analysis are shown in Figure 4B and include the signaling pathways involved in neurodegenerative pathologies such as Parkinson’s disease, Retrograde endocannabinoid signaling, Lysine degradation, Huntington disease, Alzheimer disease, Long-term depression, as well as Apoptosis, Oxidative phosphorylation, and TNF signaling pathways, which are involved in the regulation of cellular functions and are closely related to each other. Our experimental results suggest that the above-mentioned signaling pathways are the most likely potential mechanisms by which SLPQ1 promotes 5-HTP secretion in RIN-14B cells.

### 3.5. Effect of SLPQ1 on Oxidative Phosphorylation- and TNF Signaling Pathway-Related Gene Expression

**Figure 5:**
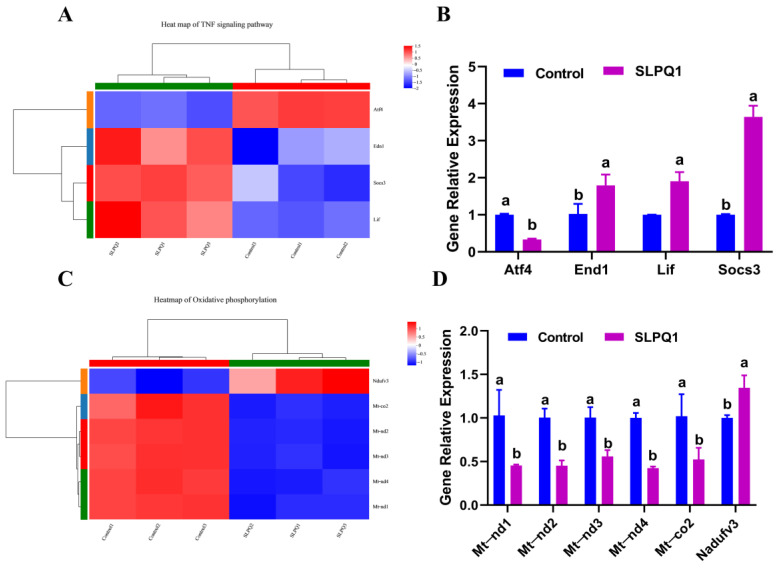
Effects of the mixture of secreted compounds, SLPQ1, collected from *Lactobacillus pentosus* LPQ1 on the genes related to the TNF and oxidative phosphorylation signaling pathways. (**A**) Heatmap of the TNF signaling pathway. (**B**) Effect of SLPQ1 on the expression of the TNF signaling pathway-related genes in RIN-14B cells. (**C**) Heatmap of the oxidative phosphorylation signaling pathway. (**D**) Effect of SLPQ1 on the expression of the oxidative phosphorylation-related genes in RIN-14B cells. Control: control group; SLPQ1: treatment group after SLPQ1. Data are expressed as the mean ± standard deviation (*n* = 3). For the same gene, two different letters (a, b) represent significant differences (*p* < 0.05).

TNF is a key mediator of inflammation, and inflammation is one of the main pathological features of depression [29,30]. Accordingly, regulation of TNF levels is considered to be important in the treatment of various Central Nervous System (CNS)-related diseases [31]. In addition, alleviation of oxidative phosphorylation was reported to improve neurological functions in mice [32]. RNA-seq analysis revealed that the *Atf4* gene was significantly downregulated, while *End1*, *Lif* and *Socs3* genes were significantly upregulated in the TNF signaling pathway in the SLPQ1 group compared with the control group. The results of the qRT-PCR expression analysis of *Atf4*, *End1*, *Lif* and *Socs3* mRNAs were consistent with the RNA-seq results, as shown in Figure 5A. Among them, the *Socs3* mRNA expression level was upregulated by 3.65-fold, and *Lif* and *End1* mRNA expression levels were upregulated by 1.91- and 1.79-fold, respectively, whereas that of *Atf4* mRNA was downregulated by 0.33-fold (Figure 5B).

The RNA-seq analysis also revealed significant upregulation of *Ndufv3* mRNA expression and significant downregulation of *mt-nd1*, *mt-nd2*, *mt-nd3*, *mt-nd4* and *mt-co2* mRNA expression in the oxidative phosphorylation pathway (Figure 5C). The results of the qRT-PCR expression analysis were similar to those detected by RNA-seq (Figure 5D). Compared with the control group, the *Ndufv3* mRNA expression level was upregulated 1.35-fold, and *mt-nd1*, *mt-nd2*, *mt-nd3*, *mt-nd4* and *mt-co2* mRNA expression levels were downregulated 0.45-, 0.45-, 0.56-, 0.42- and 0.52-fold, respectively (Figure 5D). These results indicate that the expression levels obtained by RNA-seq and qRT-PCR analyses and the upregulation and downregulation of these genes are fairly close.

### 3.6. Effects of the Active Compounds Present in SLPQ1 on 5-HTP Secretion

**Figure 6:**
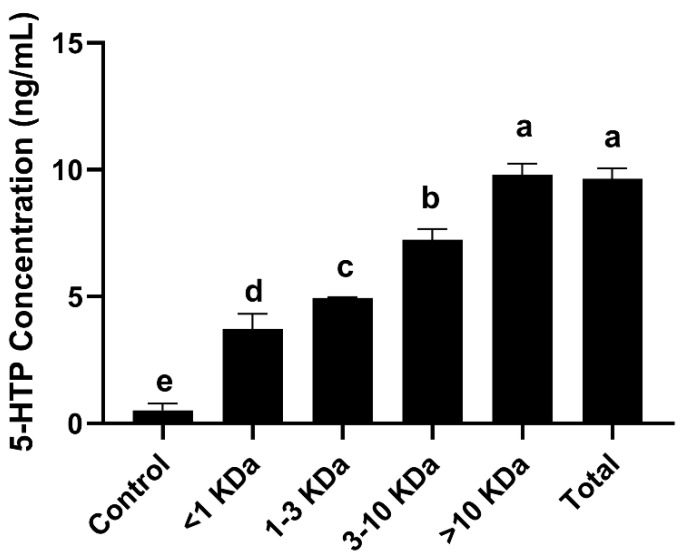
Effects of the mixture of secreted compounds, SLPQ1, collected from *Lactobacillus pentosus* LPQ1 of different molecular weights on the promotion of 5-HTP secretion. Control: control group; SLPQ1: treatment group after SLPQ1. Data are expressed as the mean ± standard deviation (*n* = 3). Different letters indicate significant differences (*p* < 0.05).

Samples were separated using ultrafiltration tubes, which through centrifugal force retain compounds of different molecular weights present in the sample. The larger the molecular weight of the compounds in the fraction of the samples, the stronger the promotion of 5-HTP production in RIN-14B cells. The highest secretion of 5-HTP was observed with fractions of SLPQ1 > 10 kDa, followed by fractions of 3–10 kDa. The results obtained clearly show that the promotion of 5-HTP secretion by SLPQ1 in RIN-14B cells is achieved mainly through fractions > 10 kDa (Figure 6).

Furthermore, the compounds larger than 10 kDa in SLPQ1 were identified using RPLC-MS/MS. As shown in Table 1, 29 proteins were found in the fractions of the samples > 10 kDa. The top five groups of compounds with relatively high contents were DUF488 structural domain protein, BspA family leucine-rich repeat surface protein, 30S ribosomal protein S5, polysaccharide biosynthesis protein and phage terminal enzyme small subunit P27 family.

## 4. Discussion

The 5-HTP is thought to play a role in mood, behavior and sleep, and is a precursor of 5-HT or serotonin, the neurotransmitter used in the treatment of depression [33,34]. Studies have found that LAB promoting 5-HTP secretion have good depression-relieving properties [9,22,23]. In addition, depression-like behavior was reported to be improved by gavage-administered 5-HTP in mice through the regulation of intestinal flora [35]. In the present study, all LAB strains were found to promote 5-HTP secretion, and it is worth noting that *L. pentosus* LPQ1 showed the highest promotion of 5-HTP secretion, reaching (9.44 ± 0.69)-fold. Remarkably, we found that SLPQ1 contained the compound through which *L. pentosus* LPQ1 exerted its 5-HTP secretion-promoting effect. The SLPQ1 concentration of 16 mg/mL was non-toxic to the cells, and the secretion of 5-HTP could reach 13.90 ± 1.06-fold.

High-throughput sequencing technology is used in RNA-seq analysis to determine the sequence of mRNA, short RNA, and non-coding RNA, which can reflect the expression level of each gene, and RNA-seq analysis is widely used as a molecular biology method to study the molecular mechanism of biological processes in treated samples [36]. This study was based on RNA-seq analysis of gene expression changes after SLPQ1 treatment in RIN-14B cells. Veen plot analysis, correlation heat map analysis and PCA plot analysis revealed that gene expression in RIN-14B cells was altered after SLPQ1 treatment, with significant inter-group differences and good intra-group reproducibility. Volcano plot analysis showed a significant effect of SLPQ1 on RIN-14B cells, with 169 upregulated and 316 downregulated genes. Cellular gene expression was changed by the effect of SLPQ1 on RIN-14B cells. The GO term enrichment analysis and KEGG pathway enrichment analysis can be used to identify cell biological functions and signaling pathways based on the DEGs identified by RNA-seq analysis, respectively. In this study, we found that the GO BP categories enriched with DEGs are mainly involved in bioregulation and metabolic processes, the GO MF categories enriched with DEGs are mainly involved in binding, catalytic activity and translation regulatory activity, and the GO CC categories enriched with DEGs are mainly associated with cellular components, organelle components and composition.

Chronic low-grade inflammation plays an important role in the pathophysiology of major depressive disorder [37]. The elevated level of serum TNF-α is considered to be one of risk assessment indicators for depression [38]. Although there are reports claiming anti-TNF-α therapy and the improvement of mood symptoms are irrelevant [39,40], some studies have proved the antidepressant effects of selective TNF-α antagonists, such as infliximab and etanercept, in non-psychiatric patients, which have been summarized in a critical review by Sarit Uzzan et al. [41]. Moreover, many animal studies have reported that TNF and 5-HT levels were negatively correlated in depressed mice, and *Lactobacillus helveticus* NS8 and *Bifidobacterium breve* CCFM1025 alleviated depression-like behavior in mice by decreasing TNF level with elevated 5-HT level [24,42]. Remarkably, excessive and prolonged stress may lead to excessive activation of ATF4, thereby inducing expression of apoptotic proteins and causing neuronal death [43]. It has also been found that increased expression of SOCS3 promotes M2 polarization in microglia, which exerts anti-inflammatory and neuroprotective effects [44]. Similarly, End-1, which is widely distributed throughout the central nervous system, is believed to control hormone and neurotransmitter release [45]. In addition, Yes-related proteins can affect astrocyte maturation and differentiation by regulating endothelial LIF secretion, which has been shown to be closely associated with episodes of major depression [46]. Interestingly, it has been shown that oxidative phosphorylation can be considered as a common pathogenesis of systemic inflammatory diseases such as ankylosing spondylitis and neurodegenerative pathologies such as dementia [47]. In this study, we found that SLPQ1 significantly elevated the expression levels of *End1*, *Lif*, *Socs3* and *Ndufv3* mRNA, and significantly downregulated the expression levels of *Atf4*, *Mt-nd1*, *Mt-nd2*, *Mt-nd3* mRNA. These findings demonstrated that the promotion of 5-HTP secretion by *L. pentosus* LPQ1 was associated with the expression of genes involved in the TNF and oxidative phosphorylation pathways.

Ultrafiltration is a widely used and practical technique for separating bioactive compounds [19]. Over the separation range, there was a positive correlation between the molecular weight of the SLPQ1 fraction and 5-HTP secretion. The effect of fractions larger than 10 kDa on 5-HTP secretion was not significantly different from the original solution (*p* > 0.05). It is slightly different from the study indicating that the 5-HTP secretion promoting compounds in RIN-14B cells are short-chain fatty acids [24]. In this study, macromolecules larger than 10 kDa in SLPQ1 and a total of 29 proteins were identified using RP-MS/MS. Zinc metalloproteinases belong to the family of matrix metalloproteinases (MMPs), which have been shown to be significantly involved in both the physiological and pathological aspects of depression, besides being important mediators of the immune response [48]. Furthermore, it has been shown that recombinant MMP-9 restores behavioral deficits manifested in learning memory in mice [49]. Flavodoxin is thought to cope with oxidative challenges, and elevated levels of oxidative stress are thought to be associated with depression [50,51]. Therefore, it can be concluded that among the 29 SLPQ1 molecules larger than 10 kDa, zinc metalloprotease (4.151%) and flavodoxin (0.004%) may be among the factors through which SLPQ1 exerts its enhancing effect on 5-HTP secretion.

## 5. Conclusions

In summary, we isolated the strain *L. pentosus* LPQ1 from the traditional Chinese fermented food Qula. *L. pentosus* LPQ1 and its secreted compounds, SLPQ1, significantly promoted the production and secretion of 5-HTP in RIN-14B cells. The promotion of 5-HTP secretion by SLPQ1 was associated with the regulation of the expression of genes involved in TNF and oxidative phosphorylation signaling pathway. Compounds larger than 10 kDa in SLPQ1 (mainly DUF488 domain-containing protein, BspA family leucine-rich repeat surface protein, and 30S ribosomal protein S5) played a key role in promoting 5-HTP secretion. The findings of this study shed light on the mechanism through which LAB strains induces 5-HTP secretion.

## Figures and Tables

**Table 1:** Identification results of compounds larger than 10 kDa in the SLPQ1 collected from *Lactobacillus pentosus* LPQ1.

Accession of Uniprot	Compounds	Relative Content (%)
A0A241RQC3	DUF488 domain-containing protein	27.441
A0A3M6KN02	BspA family leucine-rich repeat surface protein	25.096
A0A241RM09	30S ribosomal protein S5	9.968
A0A3M6LAF7	Polysaccharide biosynthesis protein	7.482
A0A3M6LTI6	Phage terminase small subunit P27 family	6.731
A0A3M6KR91	UDP-N-acetylmuramoylalanine-d-glutamate ligase	5.230
A0A241RT34	Zinc metalloprotease	4.151
A0A3M6L0D5	CDP-glycerol-glycerophosphate glycerophosphotransferase	2.603
A0A3M6LA56	Glycosyltransferase family 1 protein	2.580
A0A2K9I2H2	Metal ABC transporter substrate-binding protein	2.087
A0A2S9W1Z2	Lipid II isoglutaminyl synthase (glutamine-hydrolyzing) subunit MurT	1.848
A0A3M6KKY7	Cell surface protein	1.281
A0A3M6L8T2	ABC transporter ATP-binding protein	0.626
A0A2S9VI33	Fumarate hydratase class II	0.575
A0A3M6L2B3	Uncharacterized protein	0.558
A0A3M6L2S8	Uncharacterized protein	0.556
A0A3M6LRB4	Non-canonical purine NTP pyrophosphatase	0.227
A0A3M6LSL4	UvrABC system protein A	0.217
A0A3M6KNV5	Orotate phosphoribosyltransferase	0.150
A0A3M6LLL3	Transporter	0.131
A0A3M6LM94	ABC transporter ATP-binding protein	0.126
A0A3M6KWU4	Phage tail tape measure protein	0.078
A0A2K9HYL8	DNA polymerase	0.077
A0A3M6LFN8	Glycosyl hydrolase family protein	0.058
A0A3M6L3I3	DUF2201 domain-containing protein	0.045
A0A3M6LN80	Phosphoenolpyruvate carboxykinase (ATP)	0.039
A0A387EW54	DUF2075 domain-containing protein	0.024
A0A3M6LKY6	Uncharacterized protein	0.009
A0A2S9VNG1	Flavodoxin	0.004

## Data Availability

The data presented in this study are available on request from the corresponding author. The data are not publicly available due to privacy restrictions.

## Supplementary Materials

The following supporting information can be downloaded at: https://www.mdpi.com/article/10.3390/foods11233895/s1. Table S1: Primer sequences. Table S2: Quality of the RNA-sequence data of each sample. SLPQ1, treatment group after SLPQ1 interacted with RIN-14B cells; Control, control group.
