# Supplementary material for: Promoting Effect and Potential Mechanism of Lactobacillus pentosus LPQ1-Produced Active Compounds on the Secretion of 5-Hydroxytryptophan
Source: Foods. 2022 Dec 2;11(23):3895. doi: 10.3390/foods11233895 (PMC9740157; doi:10.3390/foods11233895)
Supplement: Supplementary file 1 [file foods-11-03895-s001.zip › foods-2008132-Supplementary.pdf]

Table S1. Primer sequences.

Target gene	Reverse (5′→3′)	Forward (5′→3′)
GAPDH	TTTGAGGGTGCAGCGAACTT	ACAGCAACAGGGTGGTGGAC
Atf4	CCAAGGAGCCGATGAGATAGTT	GACAGGCGGCAACGATACT
Mt-nd2	GGCGCCAACAAAGACTGATG	TACCCGAAGTCACCCAAGGA
Mt-nd4	GTGTGGGAAGGTTGGAGGTT	GCCAACCTAGCACTACCACC
Mt-nd3	AGGGGGAGTAGTAAGGCGAT	GACCCAACAAGTTCTGCACG
Mt-co2	AAGTGCTGGGCAAAGAATGC	CTGATGACTGCCCAACTCCC
Ndufv3	GAGGGGAGCTTTTGGACAGTG	ATAAAAAGGCGGCAGGACCAA
Mt-nd1	GAATAGGGCGAATGGTCCTG	CGAGCTCCCTTCGACTTAAC
Edn1	GAAACTGTAGAAGCCGGACA	AGCAACAGCATCAAGACCTC
Socs3	TCTTCTGCTGGGCTAACTGG	AACGGTCACCTGGACTCCTAT
Lif	GTATGGCGCAGGTGGCATT	ATGAAGGTCTTGGCCACAGG

Table S2. Quality of the RNA-sequence data of each sample. SLPQ1, treatment group after SLPQ1 interacted with RIN-14B cells; Control, control group.

Sample	Raw reads (bp)	Clean reads (bp)	Q20 (%)	Q30 (%)
Control 1	67171142	66653124	98.18	94.43
Control 2	64547394	64091508	98.17	94.39
Control 3	72493818	71963872	98.14	94.33
SLPQ1	66118396	65629050	98.17	94.43
SLPQ2	69144106	68603916	98.08	94.19
SLPQ3	73645230	73126506	98.25	94.61
